# Tuning Particle Sizes and Active Sites of Ni/CeO_2_ Catalysts and Their Influence on Maleic Anhydride Hydrogenation

**DOI:** 10.3390/nano12132156

**Published:** 2022-06-23

**Authors:** Qiuming Zhang, Xin Liao, Shaobo Liu, Hao Wang, Yin Zhang, Yongxiang Zhao

**Affiliations:** Engineering Research Center of Ministry of Education for Fine Chemicals, School of Chemistry and Chemical Engineering, Shanxi University, Taiyuan 030006, China; matthwwe@163.com (Q.Z.); lx2006294034@126.com (X.L.); lovog@163.com (S.L.)

**Keywords:** hydrogenation, particle size, maleic anhydride, Ni loading, CeO_2_

## Abstract

Supported metal catalysts are widely used in industrial processes, and the particle size of the active metal plays a key role in determining the catalytic activity. Herein, CeO_2_-supported Ni catalysts with different Ni loading and particle size were prepared by the impregnation method, and the hydrogenation performance of maleic anhydride (MA) over the Ni/CeO_2_ catalysts was investigated deeply. It was found that changes in Ni loading causes changes in metal particle size and active sites, which significantly affected the conversion and selectivity of MAH reaction. The conversion of MA reached the maximum at about 17.5 Ni loading compared with other contents of Ni loading because of its proper particle size and active sites. In addition, the effects of Ni grain size, surface oxygen vacancy, and Ni–CeO_2_ interaction on MAH were investigated in detail, and the possible mechanism for MAH over Ni/CeO_2_ catalysts was deduced. This work greatly deepens the fundamental understanding of Ni loading and size regimes over Ni/CeO_2_ catalysts for the hydrogenation of MA and provides a theoretical and experimental basis for the preparation of high-activity catalysts for MAH.

## 1. Introduction

Maleic anhydride (MA) is an important C4 fundamental material in the chemical industry that can be obtained by oxidation of coking benzene, butane, or biomass platform compounds. MA is a multifunctional and five-membered ring compound composed of one C=C, double C=O bonds, and one C–O–C functional group. A series of high-value-added fine chemicals such as succinic anhydride (SA), γ-butyrolactone (GBL), and tetrahydrofuran (THF) can be synthesized by maleic anhydride hydrogenation (MAH). These solvents and intermediates are widely used in the military, textile, pharmaceutical, and food industries [1,2,3]. The hydrogenation of MA involves C=C and C=O hydrogenation, and the investigation of hydrogenation mechanism for C=C and C=O bonds has been a hot topic. Until now, the catalysts used in the MAH have mainly been supported Ni-based catalysts, and the supports have mainly been metal or nonmetal oxides such as Al_2_O_3_, SiO_2_, TiO_2_, and CeO_2_ [4,5,6,7].

For supported catalysts, the type of active metals, the acid and base properties of the surface, defect sites, and metal–support interactions have important effects on the adsorption and activation forms, hydrogenation path, and product selectivity of MAH. Among these factors, the particle size of the active metal plays a crucial role in the catalytic performance of catalysts [8,9,10]. The geometrical structure, electronic structure, and dispersion of metal particles change dynamically with changes in particle size, and these changes lead to variation in the active sites on the catalyst surface, which significantly affects the catalytic activity of the catalyst [11,12,13,14]. Zhao et al. [15] discovered that for the Ni/SiO_2_ catalytic system, when Ni species were fine clusters, the product GBL was obtained from the hydrogenation of MA because of the strong interaction between Ni and the support. However, succinic anhydride (SA) was obtained when the Ni species was in a crystalline state and had weak interaction with the support. Li et al. [16] found that the selectivity of MAH was closely related to the grain size of the active metal, Ni. For a Ni/HY–Al_2_O_3_ catalyst, smaller sizes of Ni nanoparticles were favorable for the formation of SA, while as the Ni loading amount increased, the particle size of Ni and the selectivity of GBL increased. Meyer et al. [17] observed that NiO had a stronger interaction with the support when the Ni loading was lower (less than 8 wt%) and that the Ni nanoparticles were conducive to the generation of SA. However, when the Ni loading was gradually increased, NiO particles tended to aggregate on the surface of the support, which reduced the interaction between NiO and the support until more GBL products were finally obtained. Bertone et al. [3] found that compared with a Ni/SiO_2_ catalyst, a Ni/SiO_2_–Al_2_O_3_ catalyst had smaller grain size of Ni on the surface and showed higher GBL selectivity. They speculated that the Lewis acid on the surface of the SiO_2_–Al_2_O_3_ support promoted the formation of GBL. Ma et al. [18] prepared Pd/CeO_2_ catalysts with different Pd particle sizes on a CeO_2_ carrier and found that the CeO_2_-supported Pd single atomic catalyst showed the best activity for CO oxidation reaction. In addition, in recent works [4,19,20], we synthesized a series of Ni/CeO_2_ catalysts under different conditions and investigated deeply the important role of CeO_2_ in MA hydrogenation. These works will be very helpful for investigating the effect of the particle size and active sites of metal on MAH. On the basis of regulating the particle size and active sites of metal on CeO_2_ support, they provided a new opportunity to comprehensively understand the interaction between the active metal and support and systematically study the change in the active sites of catalysts in heterogeneous catalysis. 

Based on the above discussion, in this paper, Ni-supported catalysts with different Ni loading were prepared by the impregnation method using CeO_2_ as support, and the hydrogenation performance of the catalysts was investigated carefully. It was found that changes in Ni loading caused changes in the metal particle size and active sites, which significantly affected the conversion and selectivity of MAH reaction. In this work, the effects of Ni grain size, dispersion, surface oxygen vacancy, and Ni–CeO_2_ interaction on the hydrogenation of MA were investigated in detail, and the synthesis process of metal-supported catalysts was optimized. This paper provides a theoretical and experimental basis for the preparation of MAH catalysts with higher activity and selectivity.

## 2. Experimental Section

### 2.1. Catalysts Preparation

The chemicals, including Ce(NO_3_)_3_·6H_2_O, Ni(NO_3_)_2_·6H_2_O, and NaOH, were purchased from the Sinopharm Chemical Reagent Co., Ltd. (Shanghai, China) and used without any purification. A CeO_2_ support was prepared by the sol–gel method. First, 5.00 g Ce(NO_3_)_3_·6H_2_O was dissolved into 20 mL distilled water, and then, 6.56 g citric acid (CA) was added and stirred. After the cerium salt and citric acid were completely dissolved, the solution was heated in a water bath at 80 °C until the dry sol was formed. After drying at 120 °C for 8 h in the oven, the dry sol formed a spongy material. It was then moved to a muffle oven and calcined at 500 °C for 3 h to finally obtain the CeO_2_ support. xNi/CeO_2_ (x: mass content of Ni) catalysts with different loading contents were prepared by citric acid assisted over-volume impregnation method. For the 5Ni/CeO_2_ catalyst, 0.505 g Ni(NO_3_)_2_·6H_2_O was dissolved in a mixture of 10 mL ethanol and deionized water (volume ratio 1:1), followed by 2.00 g solid CeO_2_ and 0.139 g citric acid (mole ratio 1:1). After stirring at room temperature for 30 min, the mixture was placed in a water bath at 80 °C to volatilize the solvent. After the solvent was completely volatilized, the sample was transferred to a drying oven at 120 °C for 8 h. The obtained samples were calcinated at 450 °C for 3 h (heating rate of 3 °C/min) and then reduced for 3 h at 350 °C with H_2_ at a flow rate of 50 mL/min to prepare the catalyst, which was used for subsequent characterization and evaluation. According to different content of Ni, the catalysts were labeled as 5Ni/CeO_2_, 10Ni/CeO_2_, 15Ni/CeO_2_, 17.5Ni/CeO_2_, 20Ni/CeO_2_, and 30Ni/CeO_2_.

### 2.2. Catalyst Characterizations and Tests

X-ray diffraction (XRD) was performed using a Bruker D8 Advanced X-ray diffractometer (Billerica, MA, USA). The instrument used Cu Kα1 radiation (*λ* = 0.15418 nm) as an X-ray source and was supplied with a Ni filter and Vantec detector. The scanning range was 10~80°, and the scanning rate was 30°/min. The average crystallite size was calculated by the Scherrer formula, D = Kλ/βcosθ, where K is Scherrer’s constant (0.89). The characterizations of H_2_-TPR (hydrogen temperature programed reduction) and H_2_-TPD (hydrogen temperature programmed desorption) were determined using a Micromeritics AutoChem II 2950 chemisorption apparatus. Raman spectroscopy (Raman) was performed on a Raman spectrometer with a laser wavelength of 532 nm (HORIBA, Tokyo, Japan). X-ray photoelectron spectroscopy (XPS) was recorded using a SCIENTIFIC ESCALAB 250 X-ray photoelectron spectrometer (Thermo Company, Waltham, MA, USA) with a standard Al-Kα (h = 1486.6 eV). The spectra were calibrated according to standard C 1s (284.6 eV).

The catalytic performance of the catalyst was evaluated in a 100 mL stainless steel autoclave. First, 0.1 g catalyst was added together with 4.9 g MA and 40 mL THF into the reactor. The N_2_ was passed through to replace the air in the reactor 5 times, and then H_2_ was passed through 5 times to replace N_2_. Then, the reaction system was heated to 210 °C with stirring at 500 rpm, and the pressure was kept at 5.0 MPa. The product was analyzed using an Agilent 7890A gas chromatograph. To verify precise separation of each component in the products, the programmed temperature was selected. The primary temperature of the oven was increased to 120 °C from 100 °C at a ramp of 5 °C min^−1^, and the temperatures of the detector and injector were 190 °C and 260 °C, respectively. The conversion and selectivity of MA to the product were calculated according to the following equations [20]:

$$X_{MA}\;(\%)\;=\;\frac{C_{GBL}\;+\;C_{SA}}{C_{GBL}\;+\;C_{SA}\;+\;C_{MA}}\;\times \;100\%$$

$$S_{SA}\;(\%)\;=\frac{C_{SA}}{C_{SA}\;+\;C_{GBL}}\;\times \;100\%$$

where *C_MA_*, *C_SA_*, and *C_GBL_* represent the percent content of the reactant and the two products in the reaction, respectively, and *X_MA_* and *S_SA_* represent the conversion of MA and selectivity of SA.

## 3. Results and Discussion

### 3.1. Catalyst Characterization

**Figure 1:**
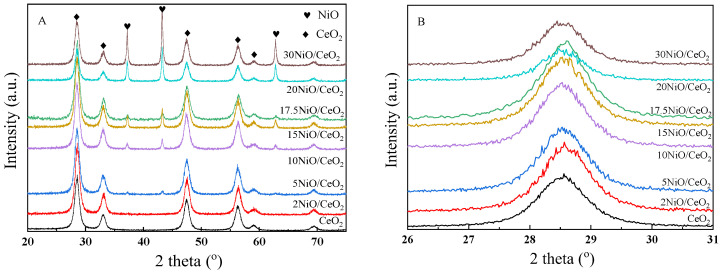
(**A**) XRD patterns of calcined xNiO/CeO_2_ samples; (**B**) the enlarged pattern at the range of 26–31°.

Figure 1 shows the XRD patterns of xNiO/CeO_2_ samples with different Ni contents. As shown in Figure 1A, after metal Ni loading, the CeO_2_ support still maintained the crystal structure of fluorite cubic phase (JCPDS File 34-0394), similarly to pure CeO_2_ [21]. The enlarged pattern (shown in Figure 1B) revealed that the diffraction peaks of the CeO_2_ (111) crystal plane in xNiO/CeO_2_ samples moved to a higher angle, indicating that the crystal cell parameters of CeO_2_ shrank after Ni loading. This may have been due to the Ni^2+^, with its smaller ionic radius (R = 0.72 nm), replacing Ce^4+^ (R = 0.81 nm) in the CeO_2_ lattice, which resulted in reductions in the cell parameters of CeO_2_ [22]. The average crystal sizes of NiO and CeO_2_ in the xNiO/CeO_2_ samples were calculated by the Scherrer formula, and the results are listed in Table 1. Compared with pure CeO_2_ support, the grain size of CeO_2_ increased after Ni loading, which may have been caused by sintering during the thermal calcination or the lattice distortion of CeO_2_ caused by Ni species [23]. 

As shown in Figure 1A, the XRD diffraction peaks at 37.0°, 43.0°, and 62.9° corresponded to the characteristic diffraction peaks of NiO’s (111), (200), and (220) crystal planes (JCPDS 47-1049), respectively. As Ni loading increased, the intensity of the NiO diffraction peak gradually increased, indicating that NiO particles aggregated on the surface of the catalyst and the grain size gradually grew. The particle sizes of NiO are also listed in Table 1, revealing that as Ni loading increased, the particle size of NiO increased from about 10.1 nm to 35.1 nm. The change in NiO grain size led to a change in the interaction between NiO and CeO_2_, which may have affected the reduction behavior of NiO and the structural difference of the catalyst surface.

**Figure 2:**
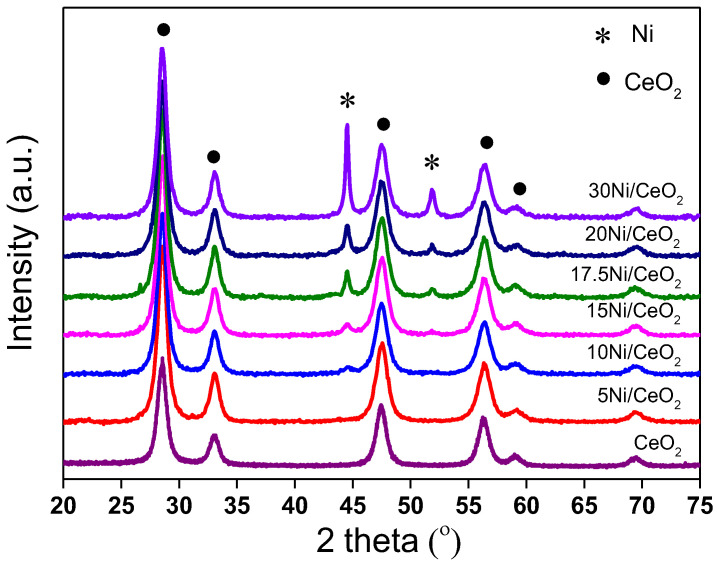
The XRD patterns of reduced xNi/CeO_2_ catalysts.

Figure 2 shows the XRD patterns of xNi/CeO_2_ catalysts after reduction at 350 °C. As shown in Figure 2, the CeO_2_ support maintained a fluorite cubic structure after reduction, and the characteristic peak of NiO disappeared, while the characteristic diffraction peak of the Ni (111) plane appeared at 44.6° (JCPDS 01-1258), indicating that NiO was reduced to metallic Ni. However, for the 5Ni/CeO_2_ catalyst, the diffraction peak of the metal Ni was not observed, which may have been due to the high dispersion of amorphous Ni species on the catalyst surface or the smaller particle size of Ni (<4 nm). The crystal sizes of Ni in xNi/CeO_2_ catalysts with different loading content were calculated by the Scherrer formula and are listed in Table 1. As the Ni loading content increased, the metal Ni aggregates on the surface of the catalyst increased, and the average grain size increased gradually from about 10.7 nm to 36.6 nm.

**Figure 3:**
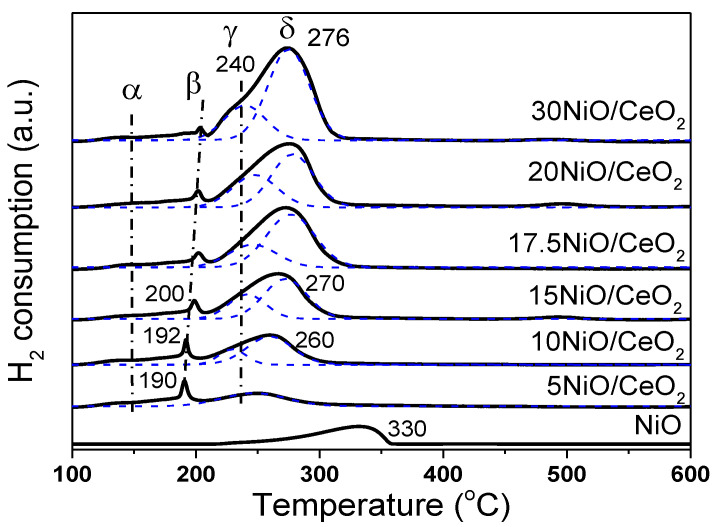
H_2_-TPR profiles of xNiO/CeO_2_ precursors.

**Figure 4:**
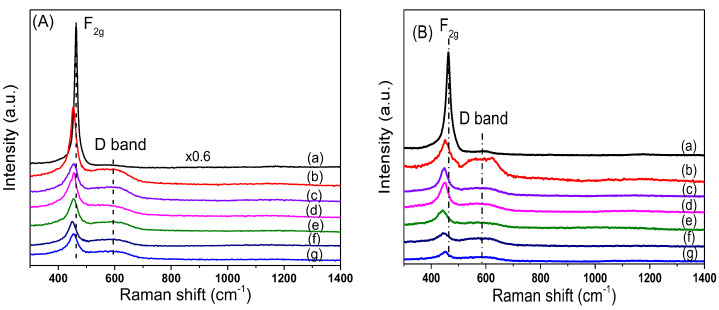
Raman spectra (**A**) xNiO/CeO_2_ precursors and (**B**) reduced xNi/CeO_2_ catalysts (a) CeO_2_, (b) 5Ni/CeO_2_, (c) 10Ni/CeO_2_, (d) 15Ni/CeO_2_, (e) 17.5Ni/CeO_2_, (f) 20Ni/CeO_2_, (g) 30Ni/CeO_2_.

Figure 3 shows the H_2_-TPR spectrum of xNiO/CeO_2_ samples. The peaks of H_2_ consumption, named α, β, γ, and δ, were well fitted through a Gauss-type function for these samples. The α peak showed lower intensity and a broader shape at about 150 °C, which was attributed to the reduction of oxygen species adsorbed on the surface of CeO_2_ [24,25]. It has been reported that parts of Ni^2+^ species could enter the CeO_2_ lattice to replace Ce^4+^, which resulted in the distortion of CeO_2_ lattice and produced oxygen vacancies to balance charges [24]. Raman results also confirmed that the loaded NiO species promoted the formation of oxygen vacancies on the CeO_2_ surface (Figure 4A). These oxygen vacancies could adsorb some small oxygen-containing molecules and generate reactive oxygen species, which can easily react with hydrogen [25]. The sharp β peak of H_2_ consumption at about 200 °C could be attributed to the H_2_ depletion caused by the dissociation and adsorption of H_2_ onto the oxygen vacancies or the Ni–Ce interface and the formation of OH groups on the surface. A similar result was found in Ni–Ce solid solution [26]. As shown in Figure 3, as Ni loading increased, the β peak gradually moves towards higher temperatures, and the peak intensity decreased, indicating that the increase in Ni content inhibited the dissociation and adsorption of H_2_ on the oxygen vacancies or the Ni–Ce interface, which may have been caused by the excessive Ni species masking the oxygen vacancies on the surface.

In general, the reduction of NiO species occurs in the temperature range of 200–300 °C. The asymmetric reduction peaks of NiO were deconvolved into two peaks for H_2_ consumption, which are labeled γ and δ, respectively. The γ peak at 240 °C was attributed to the reduction of highly dispersed NiO species closely linked to the CeO_2_ support. The stronger metal–support interaction promoted the reduction of NiO at lower temperatures [21]. The δ peak at high temperature (about 275 °C) was ascribed to the reduction of bulk NiO species aggregated on the CeO_2_ surface. From Figure 3, the reduction temperature of NiO species on the CeO_2_ surface was lower than that of bulk NiO. This was mainly because loaded NiO, with smaller size and larger surface area, could more easily contact with H_2_, which resulted in the lower reduction temperature. Moreover, oxygen vacancies and preferential reduced Ni species on the surface of CeO_2_ support (at 240 °C) promoted the dissociation and activation of H_2_, and the overflow of H atoms to NiO with large particle size was favorable to the reduction of NiO at low temperature. It should be noted that as Ni loading increased, the δ peak moved towards high temperatures. A possible reason for this is that the activation and migration of H_2_ may have been inhibited because of the increase in NiO particle size and the decrease in oxygen vacancy, thus retarding the reduction of NiO at low temperatures.

In order to study the effect of Ni loading on the surface structure of CeO_2_, Raman characterizations for xNiO/CeO_2_ samples were conducted, and the results are shown in Figure 4. The Raman peak intensity of CeO_2_ in the figure was 0.6 times that of the original peak intensity in order to facilitate comparison of results. For CeO_2_ support, a strong Raman vibration peak was observed at 466 cm^−1^, corresponding to the F2g vibration mode for the Ce–O bond in the cubic fluorite structure of CeO_2_ [27]. After the loading of NiO on the surface of CeO_2_, the F2g peak intensity of CeO_2_ decreased, the peak shape widened, and the peak position moved towards low wavelengths. This was because the strong interaction between NiO and CeO_2_ led to lattice distortion of CeO_2_, which reduced the symmetry of the Ce–O bond [25]. Besides the F2g vibration peak, the Raman vibration peak at 600 cm^−1^ was attributed to the vibration (D band) caused by defect sites on the CeO_2_ surface [25]. Compared with that of the pure CeO2 support, the peak intensity of the D band of the xNiO/CeO_2_ sample increased significantly, indicating that the existence of NiO promoted the formation of oxygen vacancies on the CeO_2_ surface. However, the vibration peak of NiO at 520 cm^−1^ could not be observed and may be covered by the F2g vibration peak of CeO_2_ [26]. Raman spectrum results for the xNiO/CeO_2_ catalyst after reduction are shown in Figure 4B. Similarly to the xNiO/CeO_2_ precursor, two Raman characteristic peaks were observed at 466 cm^−1^ and 600 cm^−1^, corresponding to the F2g vibration of Ce–O bond for cubic fluorite CeO_2_ and the D-band vibration induced by surface defects, respectively [25].

**Figure 5:**
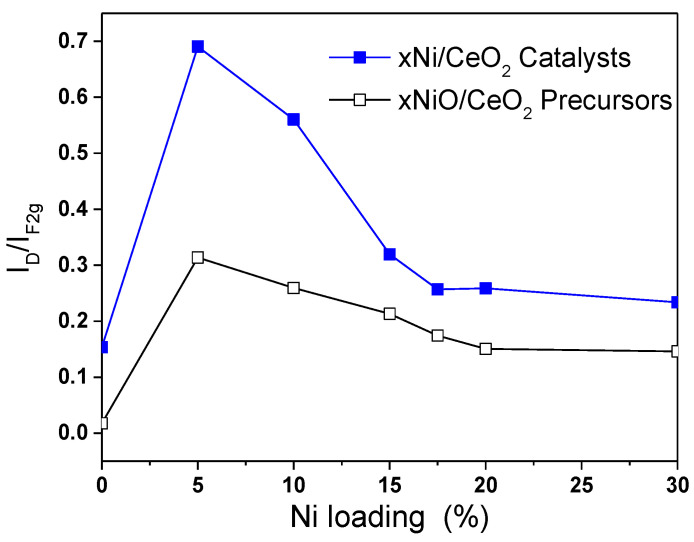
I_D_/I_F2g_ ratios for the xNiO/CeO_2_ precursors and reduced xNi/CeO_2_ catalysts.

Figure 5 shows the variation trend of the I_D_/I_F2g_ ratio with Ni content before and after reduction, which reflects the influence of Ni loading on the oxygen vacancy concentration on the catalyst surface [20]. As shown in Figure 5, the oxygen vacancy concentrations of all xNiO/CeO_2_ samples loaded with Ni were higher than that of the CeO_2_ support without Ni, which indicates that the addition of Ni was beneficial to the formation of oxygen vacancies on the surface of CeO_2_. Among these NiO/CeO_2_ samples, the I_D_/I_F2g_ ratio of the 5NiO/CeO_2_ sample is the highest, and then the I_D_/I_F2g_ ratio decreased gradually as the Ni content increased, which means that the oxygen vacancy decreased as the Ni content increased. A possible reason for this is that the aggregation of NiO and the growth in particle size on the surface of CeO_2_ weakened the interaction of NiO and CeO_2_ and covered part of the oxygen vacancies on the surface, which resulted in a decrease in oxygen vacancies.

As shown in Figure 5, compared with the xNiO/CeO_2_ samples, the I_D_/I_F2g_ ratios for the xNi/CeO_2_ catalysts increased significantly after reduction, indicating that the oxygen vacancy concentrations on the surface of the xNi/CeO_2_ catalysts increased obviously after H_2_ reduction. The oxygen vacancy increments of 5Ni/CeO_2_ and 10Ni/CeO_2_ were significantly larger than those of other catalysts with higher Ni loading, which suggests that lower Ni content was beneficial to the formation of oxygen vacancies on the surface of the catalyst. When the loaded content of Ni was low, Ni species and CeO_2_ were in close contact and interacted strongly each other, which could have promoted the reduction of the CeO_2_ surface and facilitated the formation of oxygen vacancies on the surface. However, as Ni loading increased, the active Ni species began to aggregate and cover the surface of CeO_2_, which weakened the Ni–CeO_2_ interaction and inhibited the reduction of CeO_2_ surface.

**Figure 6:**
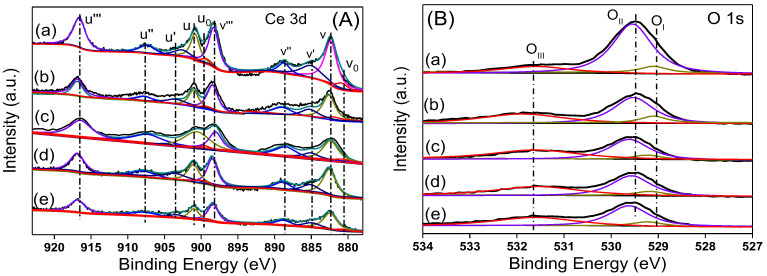
The (**A**) Ce 3d and (**B**) O 1s core-level XPS spectra of the xNi/CeO_2_ catalysts (a) CeO_2_, (b) 5Ni/CeO_2_, (c) 10Ni/CeO_2_, (d) 17.5Ni/CeO_2_, and (e) 30Ni/CeO_2_.

In order to further study the effect of Ni content on the surface species of Ni/CeO_2_, five samples of CeO_2_, 5Ni/CeO_2_, 10Ni/CeO_2_, 17.5Ni/CeO_2_, and 30Ni/CeO_2_ were characterized by the XPS technique. Figure 6A shows the Ce 3d XPS spectra of the catalyst. The peak of Ce is deconvolved into five groups of characteristic peaks according to the literature [28,29]. The three characteristic peaks labeled u and v, u’’ and v’’, and u’’’ and v’’’ belong to the XPS peaks of 3d_1/2_ and 3d_5/2_ of Ce^4+^ 3d, while the two characteristic peaks of u’ and v’ and u_0_ and v_0_ belong to the 3d_1/2_ and 3d_5/2_ of Ce^3+^ 3d. Compared with pure CeO_2_, the XPS peak of Ce^4+^ in the 5Ni/CeO_2_ catalyst moved slightly towards the high-energy direction, indicating that the strong interaction between Ni and CeO_2_ changed the electronic configuration of Ce on the surface. Similar phenomena were observed in Pt/CeO_2_ and Cu/CeO_2_ catalysts, and the peak shift of Ce^4+^ should be caused by electron transfer from metal to CeO_2_ [30,31]. According to the XPS peaks of Ce^3+^ and Ce^4+^, the concentration of Ce^3+^ on the catalyst surface was estimated, and the results are listed in Table 2. Per Table 2, the amount of Ce^3+^ on the surface of the 5Ni/CeO_2_ catalyst was the highest among these samples. As the Ni loading amount increased, the amount of Ce^3+^ on the surface gradually decreased and was even lower than that of pure CeO_2_ after reduction for 17.5Ni/CeO_2_ and 30Ni/CeO_2_. This may have been caused by excessive Ni covering the Ce^3+^ on the surface of the catalyst.

Figure 6B shows the O 1s XPS spectra of the reduced xNi/CeO_2_ catalysts. After deconvolution, three groups of XPS peaks of O were observed, representing three types of O species. O_I_ and O_II_ represented O species with different coordination in the CeO_2_ lattice, and O_III_ represented oxygen species adsorbed at defect sites on the catalyst surface. The O_I_ peak at 528.8 eV was the oxygen species coordinated with Ce^3+^ in the CeO_2_ lattice, while the O_II_ peak with slightly higher binding energy (529.4 eV) represented the oxygen species coordinated with Ce^4+^ [32]. The concentration of oxygen vacancies on the surface of the catalyst can be estimated by the ratio O_III_/(O_I_ + O_II_ + O_III_), and the results are listed in Table 2. Per Table 2, as the Ni content increased, the concentration of oxygen vacancies gradually decreased but was higher than that of the pure CeO_2_, indicating that the introduction of Ni promotes the formation of oxygen vacancies on CeO_2_ surface, which was consistent with the Raman results.

**Figure 7:**
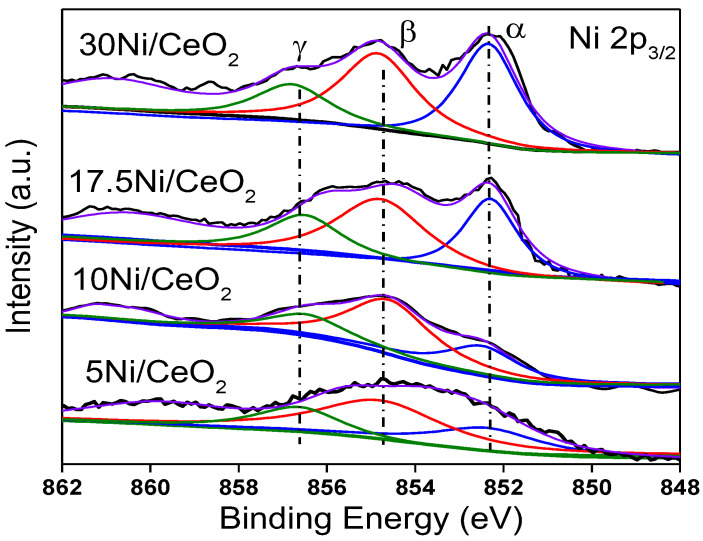
The Ni 2p_3/2_ core-level XPS spectra of the xNi/CeO_2_ catalysts.

Figure 7 shows the XPS peaks of Ni 2_p3/2_ for all catalysts. In addition to the satellite shake-up peak of Ni at about 861.0 eV, three fitting peaks represented three kinds of Ni species with different chemical states, namely α, β, and γ, which were assigned to Ni^0^ (~852.4 eV), Ni^2+^ (~854.7 eV), and Ni^3+^ (~856.8 eV), respectively. Three kinds of Ni species coexisted on the surface of the Ni/CeO_2_ catalysts. According to previous research [30,33,34], highly dispersed Ni clusters can interact with CeO_2_ support to generate the Ni–O–Ce structure, in which case the outer electrons of Ni would transfer to the 4f orbital of Ce through the Ni–O–Ce bond, which would result in the formation of Ni^2+^ or Ni^δ+^. Ni^3+^ ions should come from Ni species that enter into the CeO_2_ lattice and form a Ni_x_Ce_1−x_O_2−y_ solid solution with CeO_2_ [35]. According to the peak area of different Ni species, the proportionate relationship among different Ni species was estimated, and the results are listed in Table 2. From Table 2, as Ni loading increased, the content of Ni^0^ gradually increased, while the content of Ni^2+^ gradually decreased. This was due to the fact that when the content of loaded Ni was low, the Ni particles with smaller size were highly dispersed on the surface of the catalyst and had stronger interaction with CeO_2_ support, which made the outer electrons of Ni easily transfer to CeO_2_, thus forming more Ni^2+^. However, the increase in Ni loading led to the growth of the Ni particle size, which weakened the electron induction effect of CeO_2_ on Ni and led to the decrease in Ni^2+^ content. In addition, the relative content of Ni^3+^ was relatively low for all xNi/CeO_2_ catalysts, which means that only a small amount of Ni formed a Ni_x_Ce_1−x_O_2−y_ solid solution with the CeO_2_ support because of the limitation of the loading method (the impregnation method).

**Figure 8:**
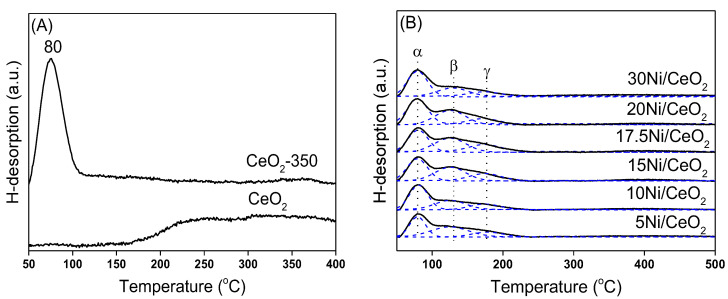
H_2_-TPD profiles of (**A**) the unreduced CeO_2_ and reduced CeO_2_ support (CeO_2_-350) and (**B**) xNi/CeO_2_ catalysts.

**Figure 9:**
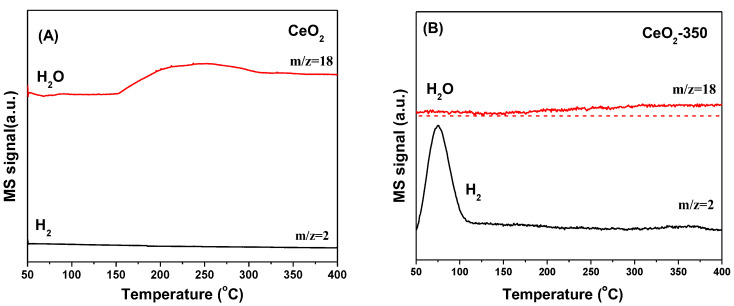
H_2_-TPD-MS profiles of (**A**) unreduced CeO_2_ (CeO_2_) and (**B**) reduced CeO_2_ (CeO_2_-350) samples.

The H_2_-TPD characterization results of the CeO_2_ support and each catalyst are listed in Figure 8. As shown in Figure 8A, the CeO_2_ support had the ability to activate and adsorb H_2_ before and after reduction at 350 °C, and the oxygen vacancies had a great influence on the form of existence for the adsorption of H_2_ [36]. In order to further study the hydrogen species on CeO_2_ surface, H_2_-TPD combined with a mass spectrometer (MS) was used to detect the desorbed H_2_ species. Figure 9A shows that H_2_ was desorbed in the form of H_2_O in the range of 150–400 °C on the surface of unreduced CeO_2_ (labelled CeO_2_), indicating that the adsorption of H_2_ on the surface was irreversible, and OH groups were generated on the surface of the support. In contrast, as shown in Figure 9B, on the surface of reduced CeO_2_ (labelled CeO_2_-350), the adsorbed atomic H was desorbed from the CeO_2_ surface in the form of H_2_ at about 80 °C, meaning that the oxygen vacancies on the surface of reduced CeO_2_ were favorable for the reversible adsorption of H_2_, which was consistent with literature reports [37].

As shown in Figure 8B, xNi/CeO_2_ catalysts with different Ni loadings had similar H_2_-TPD spectra. The desorption peak was fitted into three desorption peaks by the Gaussian method. The desorption peak (α peak) at about 80 °C was similar to the H_2_ desorption peak of CeO_2_ after reduction and was attributed to the desorption of H_2_ from the support surface. The desorption peaks β and γ were attributed to the desorption of H_2_ adsorbed on different Ni species. The β peak could be assigned to the H_2_ desorption of Ni species at the Ni–CeO_2_ interface. The strong interaction between Ni and CeO_2_ support weakened the binding ability of Ni species to H_2_ and then lowered the energy barrier of H_2_ desorption. The desorption peak γ (at 178 °C) was assigned to the desorption of hydrogen species adsorbed on the surface of Ni in bulk phase, which was similar to the H_2_ desorption on the Ni surface in Ni/Al_2_O_3_ and Ni/SiO_2_ systems and indicated that the support had little influence on the H_2_ adsorption capacity on Ni species here [38]. It can be concluded that different Ni species on the xNi/CeO_2_ surface had different adsorption and activation abilities for H_2_.

**Figure 10:**
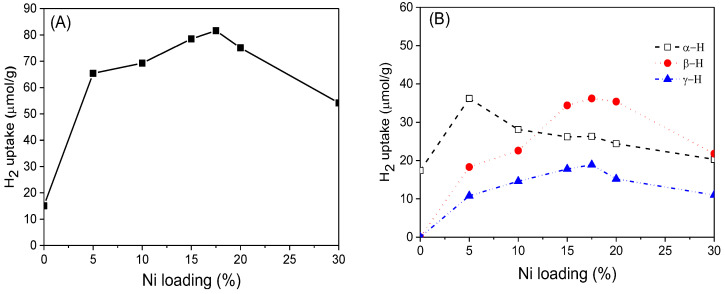
The total (**A**) and site-defined (**B**) hydrogen uptake on the xNi/CeO_2_ catalyst.

Based on H_2_-TPD results, H_2_ adsorption volumes at different active sites were estimated and correlated with Ni loading. Figure 10A shows that compared with reduced CeO_2_, the H_2_ adsorption capacity of xNi/CeO_2_ catalysts greatly increased, confirming that Ni was the center of adsorption and activation of H_2_. For all xNi/CeO_2_ catalysts, as the Ni loading increased, the amount of adsorbed H_2_ on the catalyst first increased and then decreased gradually. A possible reason for this is that as the Ni loading increased, the Ni particles aggregated on the surface of CeO_2_, and the grain size became larger, which may have reduced the number of active sites for H_2_ adsorption. Figure 10B shows that the peak area of α desorption for 5Ni/CeO_2_ was the largest among the samples. The peak area of the other samples decreased as the Ni loading increased, which indicates that the 5Ni/CeO_2_ catalyst possessed the highest concentration of oxygen vacancies for H_2_ adsorption. According to Raman and XPS results, excessive Ni was not conducive to the formation of oxygen vacancies on the surface and inhibited the ability of oxygen vacancies to activate hydrogen [39]. In addition, as the Ni loading increased, the peak areas of β and γ increased gradually in the beginning and then decreased obviously after 17.5Ni/CeO_2_. The results showed that a proper amount of Ni loading was helpful to increase the number of active sites on the support surface, while an excessive amount of Ni loading may have led to the aggregation and growth of Ni species, which could reduce the surface area of Ni particles and the number of active sites on the surface.

### 3.2. Catalytic Performance

**Figure 11:**
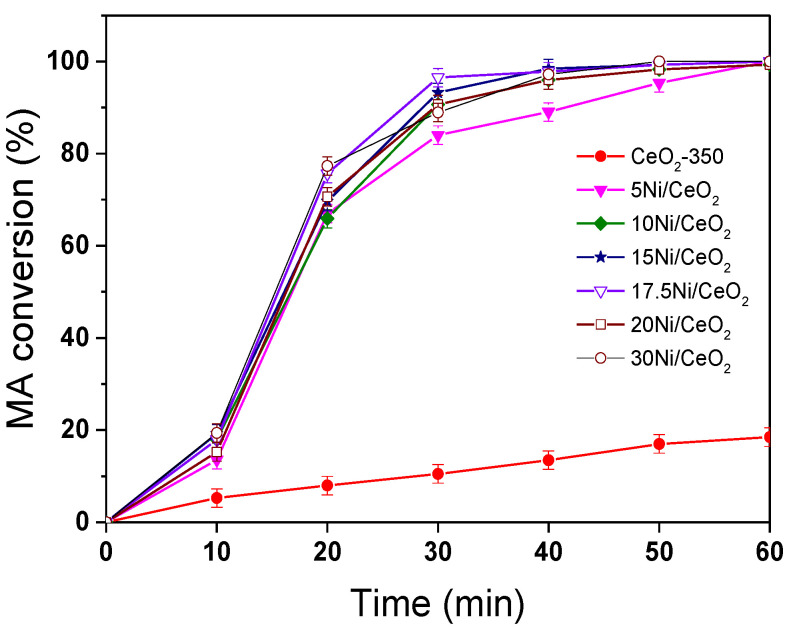
The conversion of MA on reduced the xNi/CeO_2_ catalysts.

Figure 11 shows the conversion curves of maleic anhydride (MA) over xNi/CeO_2_ catalysts and reduced CeO_2_ support at 210 °C and 5 MPa. After a reaction time of 1 h, the conversion of MA for all xNi/CeO_2_ catalysts was close to 100%, and the main product was succinic anhydride (SA), indicating that all xNi/CeO_2_ catalysts showed high hydrogenation activity for the C=C bond. It is noteworthy that the reduced CeO_2_ carrier also had a certain ability of MAH and that the conversion of MA was about 30% after 1 h under the same conditions. When Ni species were loaded on the surface of CeO_2_, the activity of MA hydrogenation increased sharply, indicating that Ni was the main active site for the MAH reaction. For all xNi/CeO_2_ catalysts, in the initial time, the catalytic activity for MAH increased gradually as Ni content increased until 17.5 wt%, and then the conversion of MA decreased slightly until 1 h.

**Figure 12:**
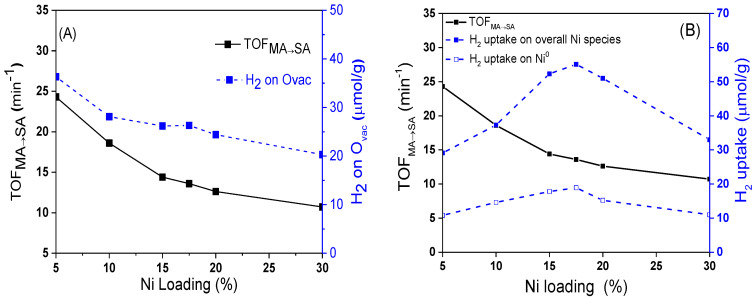
Effect of Ovac (**A**) and surface Ni species (**B**) on the TOF_MA__→__SA_ over the xNi/CeO_2_ catalysts.

In order to further investigate the C=C hydrogenation performance of xNi/CeO_2_ catalysts, the turnover frequency values for MA to SA (TOF_MA__→__SA_) over the active Ni were calculated and correlated with the oxygen vacancies, Ni species, and Ni loading content. As shown in Figure 12, the TOF_MA__→__SA_ of the xNi/CeO_2_ catalysts decreased as the Ni content increased, which was consistent with the change trend of oxygen vacancies on the surface, indicating that the oxygen vacancies of the catalyst also played an important role in the C=C hydrogenation of MA. According to H_2_-TPR and H_2_-TPD results, oxygen vacancies not only improved the dissociation and adsorption capacity of H_2_ on the catalyst but promoted the diffusion of active H on the catalyst surface, providing more active H species for the hydrogenation reaction [37]. Moreover, according to theoretical calculations, oxygen vacancies with rich electron structure can provide electrons to the active metal and enhance the electron-giving ability of the active metal, thus improving the C=C hydrogenation performance of the metal [40]. For the xNi/CeO_2_ catalytic system, it can be speculated that the synergistic effect between active metal Ni and oxygen vacancies (Ovac) could have improved the C=C hydrogenation performance of Ni.

**Figure 13:**
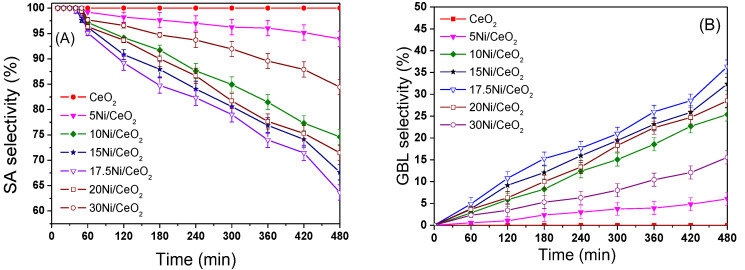
The selectivity of SA (**A**) and GBL (**B**) on reduced the xNi/CeO_2_ catalysts.

Figure 13A shows the trend of SA selectivity with reaction time on different catalysts. From Figure 13A and B, the selectivity of SA for all xNi/CeO_2_ catalysts was around 100% at the initial reaction time of 40 min and then decreased gradually while the selectivity of γ-butyrolactone (GBL) increased gradually. The selectivity of GBL on the 17.5Ni/CeO_2_ catalyst was the highest (about 35.7%) after 8 h compared with the other xNi/CeO_2_ catalysts. In addition, the selectivity of SA on the CeO_2_ support remained at 100% within 8 h of the reaction, indicating that the CeO_2_ support had almost no hydrogenation activity for the C=O bond. The above results identify that the metal Ni was the active center for the hydrogenation of SA to GBL and that the content of Ni loading significantly affected the C=O hydrogenation over the catalyst. As for the stability of the xNi/CeO_2_ catalysts, it should be noted that all samples showed good stability in the hydrogenation process. After a reaction time of 1 h, the conversion of MA for all xNi/CeO_2_ catalysts was close to 100%, and the catalysts kept their high catalytic performance. Furthermore, after five cycles of use, all the catalysts kept their high activity and selectivity, and there was no obvious decrease in either. In addition, the stability of the 17.5Ni/CeO_2_ catalyst had no obvious change compared with other catalysts in the MAH process.

It has been reported that the metal Ni with certain grain size is the active center of hydrogenation of SA to GBL. Meyer et al. [17] studied the effect of Ni loading on the hydrogenation of MA and found that Ni/SiO_2_–Al_2_O_3_ catalyst had hydrogenation activity for C=O only when Ni loading was more than 8 wt%. They concluded that a certain size of Ni grain was the active center for the hydrogenation of SA to GBL. In this work, the selectivity of GBL also showed a strong dependence on the particle size of Ni. However, when the particle size of Ni exceeded a certain amount (17.5 nm), the hydrogenation activity of C=O started to decrease. For example, though the average sizes of Ni particles on the 20Ni/CeO_2_ (18.9 nm) and 30Ni/CeO_2_ catalysts (36.6 nm) were larger than that of the 17.5Ni/CeO_2_ catalyst (17.5 nm), the selectivities of GBL were lower than that of 17.5Ni/CeO_2_ (as shown in Figure 13B).

**Figure 14:**
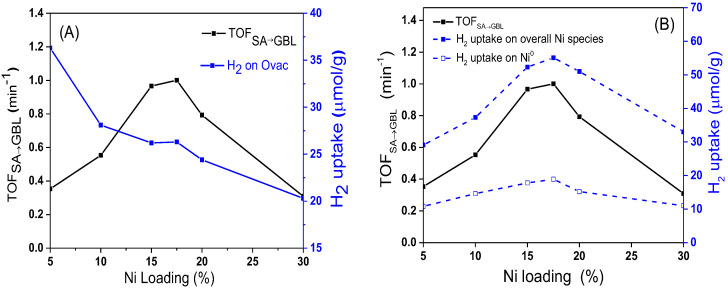
Effect of Ovac (**A**) and surface Ni species (**B**) on the TOF_SA__→__GBL_ over the xNi/CeO_2_ catalysts.

In order to understand deeply the influence of catalysts on the hydrogenation activity of C=O, the values of TOF_SA__→__GBL_ over different catalysts were calculated. As shown in Figure 14, as the Ni loading increased, the value of TOF_SA__→__GBL_ gradually increased. When the content of Ni was 17.5 wt%, the value of TOF_SA__→__GBL_ reached the maximum. It then rapidly decreased as the Ni loading increased further. At the same time, the H_2_ concentration adsorbed on Ovac decreased monotonously as the Ni loading increased. The volcanic curve for TOF_SA__→__GBL_ showed that the hydrogenation of SA to GBL was structure sensitive, which is quite different from the trend of TOF_MA__→__SA_ in Figure 12A. 

According to previous research [41,42,43], for reducible supports, such as TiO_2_ and CeO_2_, the metal–support interface is considered to be the active site of C=O adsorption activation. It was found that the C=O functional groups could be adsorbed and polarized at the interfaces of Pt–TiOx and Ni–TiOx and that the catalytic activity of hydrogenation for crotonaldehyde to crotonyl alcohol was significantly improved [41]. Because of strong interaction with the carrier, the electronic configuration for most of the metal particles at the interface was in an ionic state (such as Ni^2+^ at the interface of Ni–TiO_2_). These ionic metal particles could play the role of a Lewis acid and participate in the adsorption and activation of C=O functional groups [42]. In addition, in the hydrogenation reaction of citral, a small amount of Ni^2+^ at the Ni–TiO_2_ interface promoted the adsorption and activation of C=O in the citral molecule and finally improved the selectivity of the hydrogenation of citral to citric alcohol [41,43].

**Figure 15:**
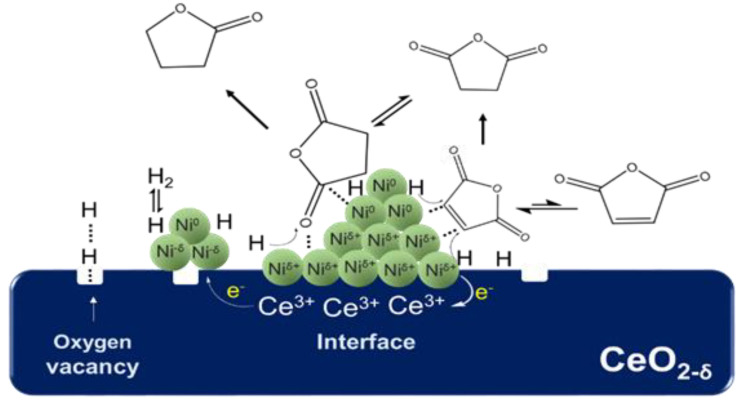
The synergy of Ni^δ+^–Ni^0^ in C=O hydrogenolysis over Ni/CeO_2_ catalyst.

As shown in Figure 14B, the change trend of TOF_SA__→__GBL_ was consistent with the change in the total amount of H_2_ adsorbed on the active sites of interface Ni and bulk Ni, which indicates that both the interfacial Ni and bulk Ni^0^ could catalyze the hydrogenation reaction of the C=O bond. According to the characterization results of H_2_-TPR, H_2_-TPD, and XPS, the Ni species at the interface showed a valence state of Ni^δ+^ because of the strong interaction with the CeO_2_ support [34]. Therefore, it can be inferred that Ni^δ+^ at the interface could also promote the adsorption of C=O on the catalyst surface as the Lewis acid site. Based on the catalytic effect of metal Ni on the adsorption and activation of C atoms in C=O and subsequent C–O bond breaking [33], we propose the possible mechanism of the Ni^δ+^–Ni^0^ synergistic effect on the hydrogenation reaction of C=O. As shown in Figure 15, first, the metal Ni^0^ adsorbs and activates C atoms in the C=O functional group, and Ni^δ+^ at the interface acts as a Lewis acid to synergistically activate O atoms. Second, the synergistic effect of Ni^δ+^ and Ni^0^ promotes the adsorption and activation of C=O, and the activated C=O group reacts with highly active hydrogen atoms on the surface of metal Ni, which results in the C=O bond hydrogenation and subsequent C–O fracture. According to this mechanism, if the particle size of Ni becomes larger, the distance between the top Ni^0^ and the bottom Ni^δ+^ increases, which weakens the synergistic activation for C=O by Ni^δ+^–Ni^0^. This constitutes a good explanation for the phenomenon in which the selectivity of GBL decreased as the average particle size of Ni increased beyond 17.5 nm.

## 4. Conclusions

In this work, Ni/CeO_2_ catalysts were synthesized by the impregnation method, and a series of xNi/CeO_2_ catalysts with different particle sizes and active sites were successfully prepared by changing the Ni loading. The effects of particle size and active sites of Ni/CeO_2_ on the hydrogenation of MA were systematically studied. It was found that the catalytic activity of the xNi/CeO_2_ catalysts was size dependent for MAH and that the metal Ni was the active center for the catalytic hydrogenation of C=C from MA to SA and of C=O from SA to GBL. In the beginning of the reaction, the hydrogenation activity of the catalyst increased as the Ni loading increased until 17.5Ni/CeO_2_ and then decreased gradually as the Ni loading increased further. The oxygen vacancies on the surface of Ni/CeO_2_ could promote the adsorption and activation of H_2_, and the synergistic effect of active metal Ni and oxygen vacancies could improve the hydrogenation of the C=C bond. The synergistic effect of Ni^δ+^ species obtained from the strong electronic attraction of the CeO_2_ support and Ni^0^ promoted the adsorption and activation of C=O in MAH. The current results confirmed that the particle size and catalytic ability of Ni/CeO_2_ catalysts could be modulated through changing the Ni loading on the CeO_2_ support. This work not only provides a deep understanding of MA hydrogenation over Ni/CeO_2_ catalysts but highlights the potential of size-dependent catalysts in heterogeneous catalysis.

## Figures and Tables

**Table 1:** The surface areas (*S*_BET_), Ni loading, and average crystallite sizes of CeO_2_, NiO, and metallic Ni in the reduced catalysts.

Sample	D(CeO_2_) (nm)	Surface Area (m^2^/g)	Ni Loading (wt%)	D(NiO) (nm)	D(Ni) (nm)
CeO_2_	11.6	35.6	-	-	-
5Ni/CeO_2_	13.4	35.8	4.8	10.1	-
10Ni/CeO_2_	13.8	37.5	10.2	21.6	10.7
15Ni/CeO_2_	13.3	34.2	15.3	24.7	15.2
17.5Ni/CeO_2_	13.8	32.1	17.2	28.2	17.5
20Ni/CeO_2_	13.7	28.4	20.5	30.5	18.9
30Ni/CeO_2_	13.1	22.2	29.1	35.1	36.6

**Table 2:** The quantitative analysis of XPS for reduced xNi/CeO_2_ catalysts.

Sample	Ce^3+^/(Ce^3+^ + Ce^4+^)	O_II__I_/(O_I_ + O_II_ + O_III_)	Ni^0^:Ni^2+^:Ni^3+^
CeO_2_	0.162	0.2066	-
5Ni/CeO_2_	0.178	0.414	0.18:0.57:0.25
10Ni/CeO_2_	0.164	0.374	0.25:0.56:0.19
17.5Ni/CeO_2_	0.146	0.364	0.34:0.45:0.20
30Ni/CeO_2_	0.139	0.334	0.43:0.39:0.18

## Data Availability

Data presented in this article are available at request from the corresponding author.
